# Characterisation of Gel-Forming Mucins Produced In Vivo and In Ex Vivo Conjunctival Explant Cultures

**DOI:** 10.3390/ijms221910528

**Published:** 2021-09-29

**Authors:** Sara I. Van Acker, Bert Van den Bogerd, Zoë P. Van Acker, Agnė Vailionytė, Michel Haagdorens, Carina Koppen, Sorcha Ní Dhubhghaill, Darlene A. Dartt, Isabel Pintelon

**Affiliations:** 1 Antwerp Research Group for Ocular Science (ARGOS), Translational Neurosciences, Faculty of Medicine, University of Antwerp, 2610 Wilrijk, Belgium; bert.vandenbogerd@uantwerpen.be (B.V.d.B.); michelhaagdorens@gmail.com (M.H.); carina.koppen@uza.be (C.K.); nidhubhs@gmail.com (S.N.D.); 2 Laboratory for Membrane Trafficking, VIB Center for Brain & Disease Research, 3000 Leuven, Belgium; zoe.vanacker@kuleuven.be; 3 Department of Neurosciences, Catholic University of Leuven, 3000 Leuven, Belgium; 4 Ferentis UAB, 02300 Vilnius, Lithuania; agne@ferentis.eu; 5 Department of Nanoengineering, Center for Physical Sciences and Technology, 02300 Vilnius, Lithuania; 6 Department of Ophthalmology, Antwerp University Hospital, 2650 Edegem, Belgium; 7 Schepens Eye Research Institute/Massachusetts Eye and Ear, Department of Ophthalmology, Harvard Medical School, Boston, MA 02114, USA; Darlene_Dartt@meei.harvard.edu; 8 Laboratory of Cell Biology and Histology, Faculty of Pharmaceutical, Biomedical and Veterinary Sciences, University of Antwerp, 2610 Wilrijk, Belgium; isabel.pintelon@uantwerpen.be

**Keywords:** conjunctiva, goblet cells, gel-forming mucins

## Abstract

One key element to the health of the ocular surface encompasses the presence of gel-forming mucins in the pre-ocular tear film. Conjunctival goblet cells are specialized epithelial cells that secrete mucins necessary for tear film stability and general homeostasis. Their dysfunction can be linked to a range of ocular surface inflammation disorders and chronic injuries. To obtain new perspectives and angles to tackle mucin deficiency, the need for an accurate evaluation of their presence and corresponding mucin secretion in ex vivo conjunctival cultures has become a requisite. In vitro, goblet cells show a significant decrease in the production and secretion of gel-forming mucins, accompanied by signs of dedifferentiation or transdifferentiation. Explant cultures on laminin-treated CLP-PEG hydrogels can, however, support the production of gel-forming mucins. Together, we challenge the current paradigm to evaluate the presence of cultured goblet cells solely based on their general mucin (MUC) content through imaging analyses, showing the need for additional techniques to assess the functionality of goblet cells. In addition, we broadened the gel-forming mucin profile of in vivo goblet cells with MUC5B and MUC6, while MUC2 and MUC6 is added to the profile of cultured goblet cells.

## 1. Introduction

The health of the ocular surface is determined by the proper functioning of all of its individual components. The ocular surface is the clinical term that includes the cornea, conjunctiva, lacrimal and accessory glands, meibomian glands, glands of Moll and Zeis, and nasolacrimal duct [1]. The primary goal of this entity is to protect the inner eye structures, while maintaining a smooth refractive surface and ensure clear vision. One key element to this is the pre-corneal and conjunctival tear film. The current concept of the tear film structure encompasses two layers that interact with one another; i.e., the muco-aqueous layer that is placed underneath the lipid layer [2,3]. The muco-aqueous layer covers the cellular glycocalyx, consisting of membrane-associated mucins, which ensure an ideal wettability and high ocular surface lubricity [4]. The gel-forming mucins are an essential part of this muco-aqueous tear film and contribute to (I) the stability of the tear film based on shear thinning properties, (II) the surface–chemical barrier that traps contaminants (e.g., pathogens, allergens, and debris), and (III) the condensation of the lipids that further stabilizes and thickens the tear film [4,5,6]. A healthy tear film is, in part, maintained by the shear thinning response to enable alternating low and high tear viscosities. To prevent damage to ocular surface epithelia, shear thinning properties enable low values of tear viscosity at high rates of stress as occurs in blinking [7]. High viscosity, on the other hand, takes place at low shear stress levels in open eyes to maintain a continuous tear film [7]. 

As a result of the pivotal role mucins play in maintaining the tear film, it is not surprising that the number and functionality of mucin-producing conjunctival goblet cells are altered in various ocular surface disorders. Hypersecretion and hyperplasia of goblet cells are characteristic of allergic conjunctivitis, chronic injuries, conjunctival papilloma, and pterygium [8,9]. In contrast, an overall decrease in goblet cell function and number is found in inflammatory disorders, such as dry eye disease, Stevens–Johnson Syndrome, Sjogren’s syndrome, and graft versus host disease [8]. The role of mucins in the vicious circle of ocular inflammatory disorders has become well-defined over the last years, leading to renewed interest in research on mucin deficiency (reviewed in [10]). 

Conjunctival goblet cells are the main source of gel-forming mucins on the ocular surface and secrete, aside from mucins, trefoil factors into the tear film [11,12,13]. To date, five gel-forming mucin (MUC) family members have been identified, of which three have been localized to the conjunctiva, an epithelium of the ocular surface: MUC2, MUC5AC, and MUC19 [10,14]. Gel-forming mucins are large, heavily O-glycosylated proteins that are characterized by specific cysteine-rich domains at both the N and C terminus to enable homo-multimerization and, consequently, gel formation [10]. Structurally, mucins are composed of a polypeptide backbone containing threonine- and serine-enriched repeat domains that are used as anchor points for O-glycosylation. The sugar side chains are, in turn, responsible for the specific bottle brush structure [10,15]. Gel-forming mucins not only interact with one another but also with trefoil factors. These proteins are known binding partners for the gel-forming mucins, thereby modulating the physical properties of the mucus and rheology of the tear film [16,17]. The functions of the gel-forming mucins are derived from studies that focused on MUC5AC (e.g., a MUC5AC knockout mouse model, serving as dry eye model [18]), which is the most prevalent secreted mucin at the ocular surface and a well-known goblet cell marker [13].

To develop new approaches to treat mucin deficiency and to determine the as yet undefined in vitro requirements of conjunctival goblet cells [19], a proper evaluation of their presence and corresponding mucin secretion in ex vivo cultures is needed. Goblet cells are currently distinguished from isolated or cultured stratified squamous epithelial and stem cells based on their storage of mucin, visualized by Periodic acid-Schiff (PAS)-staining or lectin binding, and/or their selective MUC5AC production [20,21,22,23,24,25]. In this study, we aimed to challenge the established paradigm that simply staining goblet cells as standard assessment procedure is sufficient, since the presence of mucin is not correlated with production capability and, as such, does not reflect a healthy conjunctival goblet cell. Our goal was, therefore, to study the functionality of the goblet cells in vivo and in vitro. We assessed ex vivo conjunctival cultures established on culture plastic ware and extracellular matrix hydrogels, using additional assays to specifically examine mucin production, storage and secretion in culture over time, in the hopes of representing true goblet cell function.

## 2. Results

### 2.1. Change in Intracellular Mucin Storage and Secretion during Two Weeks of Culture 

**Figure 1:**
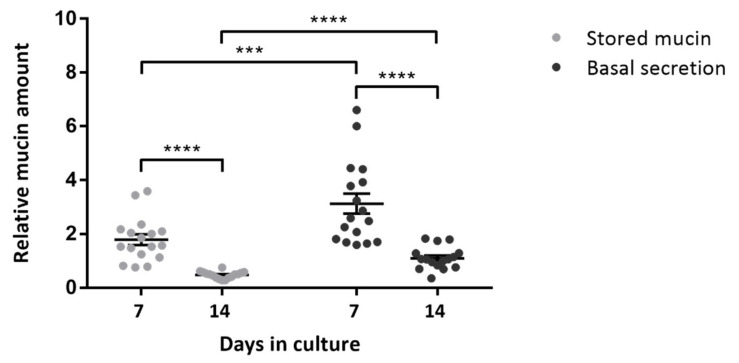
Goblet cells show a high mucin-producing capacity that decreases with time in culture. Change in normalized mucin amount stored (grey points) or secreted (black points) from the conjunctival outgrowth at 7 or 14 days in culture. Each bar represents the mean ± standard error of the mean (SEM) of technical replicates from three donors (*n* = 5 for 2 donors, *n* = 6 for 1 donor). Evolution of the stored mucin and basal secretion is analyzed with an unpaired *t*-test and Mann–Whitney test, respectively. Differences in the stored and secreted compartment at 7 and 14 days in culture are investigated using the Wilcoxon and paired *t*-test, respectively. ***, *p*-value = 0.001; ****, *p*-value < 0.0001.

To assess the functionality of cultured goblet cells in a conjunctival outgrowth, the normalized intracellular mucin storage and secretion was determined 7 and 14 days after culture initiation (Figure 1). A downward trend is observed when looking at the evolution of stored mucins (Figure 1, unpaired *t*-test, *p*-value < 0.0001) or basal secretion (Figure 1, Mann–Whitney test, *p*-value < 0.0001) during the culture period. In contrast, the basal mucin secretion remains elevated over the intracellular storage levels at 7 days in culture (DIC) (Figure 1, Wilcoxon test, *p*-value = 0.0001) and 14 DIC (Figure 1, paired *t*-test, *p*-value < 0.0001).

### 2.2. Gel-Forming Mucins mRNA Expression 

**Figure 2:**
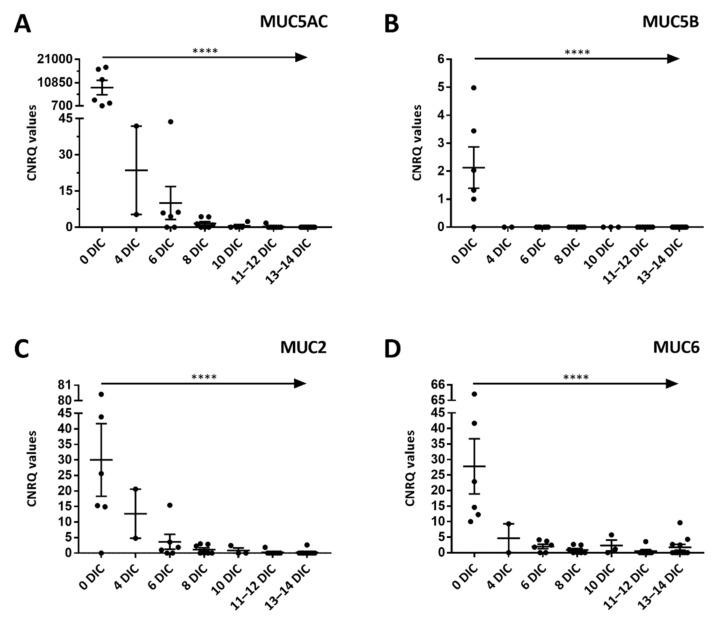
mRNA expression of gel-forming mucins during a two-week culture period. Transcript levels of cells enzymatically removed from conjunctival tissue and cells from conjunctival explant cultures are normalized and set against the lowest expression levels found in all the samples for each gene separately to obtain the Calibrated Normalized Relative Quantity (CNRQ) values. Quantifications were performed for mucin (MUC)5AC (**A**), MUC5B (**B**), MUC2 (**C**), and MUC6 (**D**) on 0–14 days in culture (DIC). Each dot represents a CNRQ value of a different donor at each specific time point. Data are depicted as the mean ± SEM and fitted in a mixed effect model was *p*-value < 0.0001 (****) for each type of mucin.

To determine if mRNA expression declines similarly to mucin storage and secretion, we assessed the mRNA expression of four secreted, gel-forming mucins; MUC5AC, MUC5B, MUC2, and MUC6 (Figure 2). The Calibrated Normalized Relative Quantity (CNRQ) values depicted in the graph represent the normalized, relative expression levels for each mucin gene separately. These values are acquired through the normalization with four reference genes and their relation with the lowest expression level of a specific gel-forming mucin gene in all the samples [26]. As shown in Figure 2, the relative mRNA expression of the four mucins drops significantly with time in culture (Fixed effect test of mixed effect model, *p*-value < 0.0001). We were unable to detect MUC5AC mRNA in the majority of samples after 8 DIC and the transcript completely disappeared at the end of the two-week culture period (Figure 2A). The magnitude of the dramatic decline is demonstrated in view of the relative expression levels in lysed conjunctival cells that are on average up to 9000 times more elevated (Figure 2A, 0 DIC), while only magnitudes of 4.4 are found after 8 DIC (Figure 2A). A substantial decline is also observed for MUC5B. MUC5B is only detected in primary lysed conjunctival cells and not in cultured samples (Figure 2B, 0 DIC). Expression of MUC2 and MUC6 can be detected in some samples throughout the culture period, in contrast to MUC5AC and MUC5B levels (Figure 2C,D). The MUC2 and MUC6 mRNA levels of in vivo conjunctival cells are also of comparable elevated magnitude, i.e., 30.01 (MUC2) and 24.37 (MUC6), when set against the lowest mRNA quantity found in all samples (Figure 2C,D). However, some small differences can be seen between the courses of expression levels over time. Despite of higher MUC2 relative levels being found until 6 DIC, the expression drops to zero (or below the detection limit) in the majority of the samples afterwards, while MUC6 mRNA can be detected in relatively more samples (Figure 2D). 

### 2.3. Protein Detection of MUC2, MUC5AC, and MUC6

**Figure 3:**
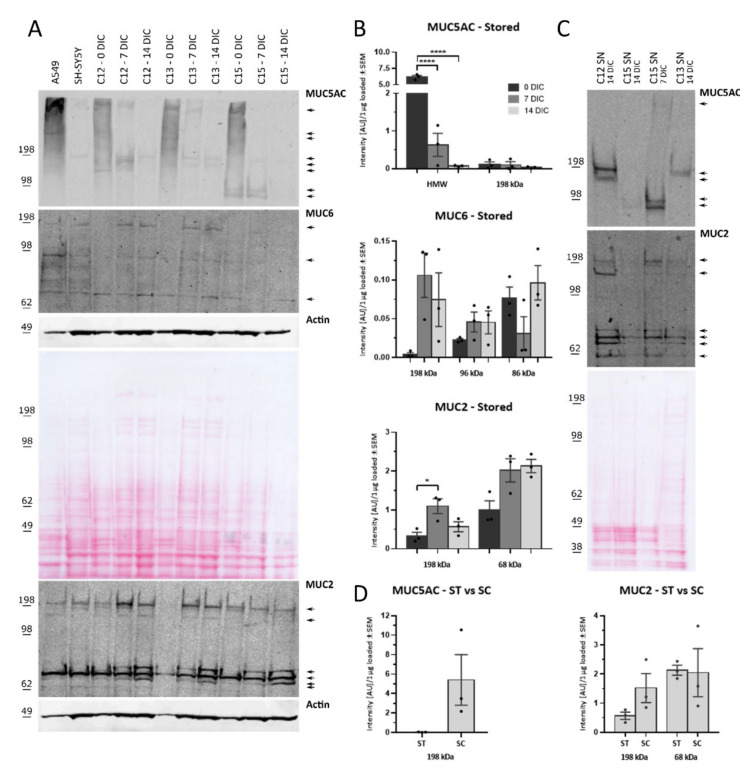
Analysis of molecular weight and intensity of protein bands for each mucin in cellular extracts and secretomes. WB analysis of gel-forming mucins in (**A**) 12 µg of cell extracts and (**C**) 2.2 µg cell culture supernatant (SN). (**A**) β-actin and a ponceau staining (illustration of a representative gel) were used as internal controls for the intracellularly stored mucins and (**C**) a ponceau staining for the centrifuged SN. A uniform loading was achieved across all samples, with the exception of donor 2 (C2, 0 days in culture (DIC)) in the MUC2 blot, as only limited material was available for this donor. (**B**) The change in bands of different of molecular weight from in vivo levels to their amount after 7 and 14 DIC was evaluated based on the changes in normalized intensity per µg loaded protein (AU, arbitrary units). HMW stands for high molecular weight and represents the MUC5AC smear at the top of the gel in A. One-way ANOVA with Tukey’s multiple comparison test or Kruskal–Wallis test was used. * *p*-value < 0.5; **** *p*-value < 0.0001. (**D**) Difference in the amount of stored (ST) and secreted (SC) molecular weight isoforms of MUC5AC and MUC2 at 14 DIC. The paired *t*-test was used for statistical analysis. The intensity data of (**C**) and (**D**) are depicted as mean ± SEM and each dot represent the normalized intensity per µg loaded protein for donor C12, C13, and C15.

We assessed the presence of cellular (stored) and secreted MUC2, MUC5AC, and MUC6 by western blot analysis to compare with the enzyme-linked lectin assay (ELLA) results and establish a transcript-protein level correlation. The A549 and SH-SY5Y cell lines were loaded as reference (Figure 3). All three gel-forming mucins—MUC2, MUC5AC, MUC6—are present as different molecular weight species. Intracellular MUC5AC exists under eight different forms (Figure 3A, arrows). The upper most band is seen as a smear, corresponding to the monomeric form (i.e., around 500 kDa [27]) with gradually increasing glycosylation patterns. The lower bands (around 98 and 198 kDa) likely represent cleavage products with different glycosylation patterns. The intensity of the ~500 kDa smear significantly decreases when the in vivo levels are compared to 7 DIC and 14 DIC (Figure 3B, one-way ANOVA, *p*-value < 0.0001). The time-effect on the lower band’s intensity is less explicit (Figure 3B, one-way ANOVA, *p*-value = 0.6096). MUC2 and MUC6 show a different profile compared to MUC5AC. No bands can be observed at the predicted molecular weights of MUC2 (i.e., around 600 kDa [27]) and MUC6 (i.e., around 400 kDa [27]), but rather appear as different lower molecular weight forms (Figure 3A). We were unable to establish a clear cleavage pattern for MUC6 as only three single bands were identified at considerably different molecular weights: ~198 kDa, ~90 kDa and ~70 kDa. In contrast to MUC5AC, the intensity of the three MUC6 bands per µg loaded protein did not significantly change over time (Figure 3B; 198 kDa, Kruskal–Wallis test, *p*-value = 0.0714; 96 kDa, one-way ANOVA, *p*-value = 0.3262; 86 kDa, Kruskal–Wallis test, *p*-value = 0.2964). Despite not being significant, the MUC6 isoforms at 198 kDa are remarkably more present in cultured cells compared to lysed cells for two donors (Figure 3B). MUC2 has an upper band around 198 kDa and a band at around 68 kDa (Figure 3A). During culture, two extra bands appear, close in molecular weight to the 68 kDa band. It is, therefore, plausible that these two bands represent changes in glycosylation pattern. The addition of the two bands results in a higher relative intensity. However, no significant changes could be observed with duration of culture time (Figure 3B, Kruskal–Wallis test, *p*-value = 0.1321). In contrast, we did find a significant higher intensity of the 198 kDa bands between 0 DIC and 7 DIC (Figure 3B, one-way ANOVA with Tukey’s post-hoc, *p*-value = 0.0215), which nonetheless did not remain. Hence, unlike the MUC5AC gene-protein correlation, MUC2 and MUC6 do not correlate well. 

Next, the extracellular (secreted) mucin levels in the lysed centrifuged apocrine secretion pellet were investigated (2.2 µg protein loaded, Figure 3C). We found a similar cleaved/glycosylated pattern for stored and secreted MUC2, while less cleaved/glycosylated bands are found for secreted MUC5AC compared to its stored counterpart (Figure 3A,C). When the difference in intensity between the visible intracellular and secreted molecular weights bands was assessed, no significant changes could be detected (Figure 3D). The average intensity of the MUC2 bands at 68 kDa is almost identical (Figure 3D, 2.13 (stored) vs. 2.05 (secreted); paired *t*-test, *p*-value = 0.9142). In contrast, the average intensities of the 198 kDa MUC5AC isoform are quite distinct (Figure 3D, 0.03 (stored) vs. 5.41 (secreted); paired *t*-test, *p*-value = 0.1736) as well as those of MUC2 (Figure 3D, 0.57 (stored) vs. 1.52 (secreted); paired *t*-test, *p*-value = 0.1519). This observation is in line with our finding that secretion amounts for a substantial part of the total mucins stored by the cells (Figure 1). 

### 2.4. Morphological Identification of Cultured Conjunctival Goblet Cells 

**Figure 4:**
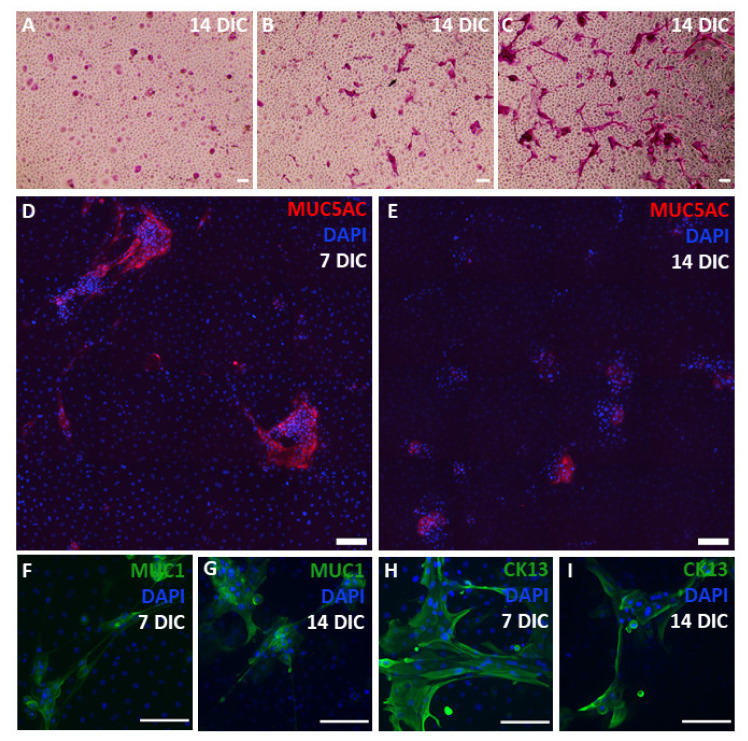
Morphology and organization of the goblet cells in ex vivo explant cultures. Representative images of ex vivo conjunctival explant cultures at 7 and 14 days in culture (DIC) of four different donors. Top row are PAS-stained cells (**A**–**C**), while the middle and bottom row show immunocytochemical illustrations (**D**–**I**). The investigated markers and corresponding colors are depicted on the images. Nuclei are counterstained using DAPI. Scale bar = 100 µm.

The mucin production and secretion of the cultured goblet cells is found to be compromised. Hence, we examined if the morphology and organization of the goblet cells in ex vivo explant cultures after 14 DIC was also affected using a non-specific staining with PAS (Figure 4A–C). A considerable amount of PAS-positive cells was observed in the different cultures, which would indicate mucin production to be not impaired (Figure 4A–C). However, we identified two morphological distinct PAS-positive cell types; (I) round, solitary cells of different sizes and (II) elongated cells with considerable extensions that have the tendency to cluster (Figure 4A–C). Explant cultures of the same donor showed parts with dominant round cells (Figure 4A) or elongated PAS-positive cells (Figure 4C) as well as a combination of both types (Figure 4B). As it was shown recently by García-Posadas et al. that immunocytochemistry is a sensitive method to demonstrate MUC5AC [28], we questioned whether we could find the same PAS-morphologies through immunocytochemical staining for MUC5AC (Figure 4D,E). Throughout the culture period, both round and elongated MUC5AC-positive cells could be observed (Figure 4D,E). However, there seems to be a change from large, intensely stained groups of MUC5AC-positive cells (7 DIC, Figure 4D) towards smaller, less intensely stained groups (14 DIC, Figure 4E). This visually perceived loss of intensity in combination with the smaller groups and seemingly fewer single MUC5AC-positive cells with increased DIC could correspond with the diminished stored MUC5AC content, detected using western blot (Figure 3), and the decreased mucin storage, determined with ELLA (Figure 1). Our observation that MUC5AC-positive cells could still be observed after 14 DIC, while almost no MUC5AC protein could be detected using western blot, is in line with the results of García-Posadas and colleagues [28]. The unexpected elongated morphology and compromised functionality led to the hypothesis that the ex vivo culture conditions are not able to support differentiated goblet cells and that they dedifferentiate or transdifferentiate. As conjunctival epithelial cells and goblet cells originate from the same bipotent stem cell [29], we stained the conjunctival cultures for two common stratified squamous epithelial cell markers, i.e., MUC1 and the intermediate filament protein cytokeratin (CK) 13 (Figure 4F–I). The single and grouped elongated cells indeed show positivity for MUC1 and CK13 at 7 and 14 DIC (Figure 4F–I), reminiscent of dedifferentiation or transdifferentiation towards a stratified squamous epithelial phenotype. 

### 2.5. Substrate-Based Culture Technologies to Improve Goblet Cell Functionality

**Figure 5:**
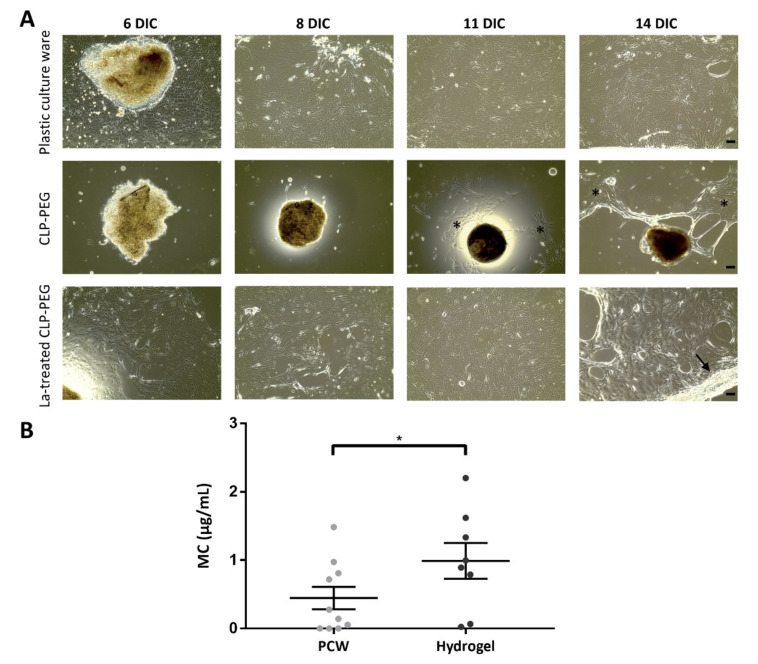
Comparison of conjunctival outgrowth and mucin secretion on plastic culture ware and CLP-PEG hydrogels. (**A**) Representative pictures of explant outgrowth of the same donor plated under three conditions; plastic culture ware, CLP-PEG hydrogels, and laminin-treated CLP-PEG hydrogels. Cultures were imaged at the indicated time points during the culture period. Asterisk = small outgrowth on CLP-PEG scaffolds, black arrow = reflective border, scale bar = 100 µm. (**B**) Comparison of absolute mucin concentration of explant cultures on plastic culture ware (PCW) and laminin-treated CLP-PEG scaffolds. Both conditions had the same culture time and the growth surface of the 24-well plate (i.e., 1.9 cm^2^) is similar to the one of the scaffold (i.e., 1.8 cm^2^). Each bar is the mean ± SEM of technical replicates (*n* = 3, grey dots for PCW cultures, black dots for hydrogel cultures) from three donors, except for one donor that has 4 and 2 replicates for PCW and laminin-treated scaffolds, respectively. Statistical significance was defined with a paired *t*-test. * *p*-value < 0.5.

As such, we were interested in identifying crucial elements in the conjunctival explant culture protocol that could improve mucin production and secretion. We explored extracellular matrix scaffolds, more specifically hydrogels consisting of collagen-like-peptides (CLP) conjugated to polyethylene glycol (PEG) maleimide. We were, however, unable to obtain much conjunctival outgrowth on these CLP-PEG hydrogels (Figure 5A). As some conjunctival cells could attach to the surface and give rise to small cell populations (Figure 5A, asterisks), we hypothesized that the problem did not arise from the mechanical properties of the hydrogel but rather from the conjunctival cells experiencing difficulties to attach to the hydrogel after migration out of the explant. We therefore chose to treat the CLP-PEG scaffolds with laminin, which is a well-known component of the conjunctival basement membrane [19]. The presence of laminin did facilitate conjunctival cell attachment and migration. Similar growth patterns could be observed between explants plated on plastic culture ware and laminin-treated CLP-PEG hydrogels (Figure 5A). Confluent cultures were obtained after a two-week culture period as demonstrated by the reflective border, indicated by an arrow, in Figure 5. To determine if the use of laminin-treated CLP-PEG hydrogels would enhance mucin secretion, a comparison was established with the basal secretion of conjunctival explant cultures on plastic culture ware using the ELLA assay (Figure 5B). We looked at the absolute mucin concentrations found in the supernatant. Laminin-treated hydrogels indeed improve mucin secretion, with secreted levels in scaffold-grown cultures increasing from 0.43 µg/mL on plastic culture ware to 0.97 µg/mL on laminin-treated hydrogels (Figure 5B; paired *t*-test, *p*-value = 0.034).

## 3. Discussion

Goblet cells represent a conjunctival population of gel-forming mucin-producing cells that are crucial for maintaining a healthy tear film and therefore ocular surface [4,5,6]. Their central role is emphasized by their dysregulated numbers and mucin production in various ocular surface disorders [10]. It was not until recently that more data were published regarding the life-cycle of goblet cells, their role in immunoregulation and the regulation of mucin expression and secretion [10]. Understanding these factors is imperative to develop new therapeutic strategies to target mucin deficiency and regain ocular surface homeostasis upon pathology. Furthermore, the cultivation of goblet cells could open new therapeutic strategies to restore mucin-deficient, damaged conjunctival tissue in severe ocular surface disorders [10,19,30]. In this study, we investigated the functionality of the goblet cells more thoroughly in ex vivo conjunctival cultures to challenge the standard practice of characterizing goblet cells using only staining procedures. 

We found a diminishing gel-forming mucin secretion over a two-week culture period, possibly attributed to mucin secretion of goblet cells in the native conjunctiva being under strict regulation, e.g., by the parasympathetic and sympathetic innervation [31]. It is, therefore, plausible that the removal of goblet cells from their natural surroundings into a culture environment, lacking physiological stimuli, could explain why goblet cell secretion, which we link to their differentiation status, could not be maintained. As some MUC5AC-positive cells are still observed after 14 DIC, we hypothesize that the cultured goblet cells dedifferentiate into a precursor goblet cell state with only limited MUC5AC production. Another explanation could be that transdifferentiation occurs during the culture period. Transdifferentiation, i.e., a process which is distinctive from the usual differentiation pathway where one differentiated cell type transforms into another one, has already been reported in explant cultures from swine trachea epithelium [32]. We found the same uncharacteristic elongated morphology of PAS-positive cells in MUC1-, MUC5AC-, and CK13-positive cells. MUC1 and CK13 are markers for conjunctival stratified squamous epithelial cells [33,34], which supports the hypothesis that our cultures have an intermediate elongated cell type with both goblet and stratified squamous epithelial cell characteristics. Of note, both cell types result from a bipotent conjunctival stem cell [29]. We thus describe a previously unreported and crucial point of interest. While PAS- and MUC5AC-positive cells might still be present at the end of a culture period, we demonstrate that these cells are not necessarily as functionally active as in primary tissue. We detected a decrease in mucin secretion as well as signs of de- or transdifferentiation. These findings represent the foundation to challenge the standard characterization practice that assesses cultured goblet cells by simple mucin staining. The implementation of functional assays such as qPCR, ELLA, and western blot seem imperative to determine true well-functioning goblet cells ex vivo.

In search of potential culture stimuli to maintain and support gel-forming mucin secretion, we tested the use of CLP-PEG scaffolds. CLPs are small peptide units that self-assemble into triple helical nanofibers, such as collagen [35]. A soft hydrogel is obtained through the conjunction of CLP to a PEG backbone [35]. Crosslinked CLP-PEG hydrogels are compatible with ocular surface epithelia. We previously demonstrated their use as culture substrate for limbal epithelial stem cells [36]. Islam and colleagues further showed that CLP-PEG hydrogels have potential as corneal implants. The hydrogels were stably integrated in a mini-pig animal model, in which it also promoted corneal-, stromal-, and nerve regeneration [37]. Despite the biocompatibility with corneal and limbal epithelium, this study only observed a limited conjunctival outgrowth on the hydrogels. Hence, we treated the CLP-PEG hydrogels with laminin. Laminin is one of the structural proteins assembling the conjunctival basement membrane alongside different collagen types, fibronectin, thrombospondin 4, and other glycoproteins. We showed that laminin treatment led to a similar morphological conjunctival outgrowth as compared to the explant cultures initiated on plastic culture ware. Nevertheless, these treated CLP-PEG hydrogels had an effect on the gel-forming mucin production as an enhanced secretion is observed. We have yet to define if the enhanced mucin secretion is due to (I) the laminin-coating itself, (II) the intrinsic characteristics of the CLP-PEG scaffold (e.g., elastomechanics) in which case laminin would only enable cellular attachment to the CLP-PEG scaffolds (cfr. no outgrowth was observed without the laminin-coating) or (III) a combination of both factors.

Given the essential contribution of secretory mucins to the stability of the human ocular surface, we further characterized the range of mucins secreted by goblet cells in both primary tissue and explant cultures. Most studies on the ocular surface have focused on MUC5AC. Hence, MUC5AC is not only the most abundant mucin in the ocular surface, but also the best characterized one [10]. Other mucins include MUC2, MUC5B, and MUC6, in order of characterization level. MUC5AC has been described to be exclusively produced and secreted by conjunctival goblet cells and its transcript levels reach approximately 5.6 × 10^4^ molecules per microgram of RNA, which is 10-fold lower as compared to those of β2-microglobulin [10,38]. MUC5AC levels are further regulated on the post-transcriptional level, with protein levels ranging from undetectable to more than 200 µg/mL in tears of healthy individuals [39]. Aside from Schirmer strip testing, other studies have demonstrated MUC5AC presence in conjunctival tissue as well as tears, using either SDS-polyacrylamide gels [38,40] or SDS-agarose gels [41]. Several MUC5AC molecular weight bands are found, which are partly attributed to the intracellular mucin synthesis and transport process where the polyprotein backbone undergoes several glycosylation rounds, oligomerization steps, and possible cleavages [42,43,44,45,46,47]. Different studies detected positive smears at the top of the gel [38,41], a minor smear above 207 kDa with two corresponding bands [40], and lower bands between 20 and 85 kDa in human conjunctival extracts [38]. Mckenzie et al. found similar patterns in tears, while Spurr-Michaud et al. demonstrated a lower smear at 250 kDa. MUC5AC has also been detected in primary cultures of human conjunctival tissue; however, only one band was reported, without the corresponding kDa marker [48]. We expanded the analysis from isolated tissue samples to explant cultures. Similarly, we detected a variety of molecular weights forms, ranging from a high molecular weight smear of over 198 kDa to smaller fragments of ~98 kDa. The reason underlying the differently reported molecular weights requires additional characterization studies to define the protein structure and glycosylation pattern of the different bands. 

Mucins may further be processed post-secretion, with studies describing the occurrence of more sparsely glycosylated and, therefore, smaller monomers in the extracellular milieu [41,49]. However, while theoretically possible, our data do not support the actual occurrence of post-secretory processing of MUC5AC. We detected the same low molecular weight bands in intracellular fractions as in the culture’s supernatant. Of note, as proteinase inhibitors are used in most studies, including this one, it is unlikely that the observation of different molecular weight forms results from product breakdown occurring after cell lysis. We also note that most studies on human tissue are restricted in the amount of samples that can be included. As a variable number tandem repeat polymorphisms exists for *MUC2*, *MUC5AC* and *MUC6* [50], these may account for additional inter-individual (and study) differences. The effect could be especially of note for MUC2 and MUC6, for which up to two-fold differences in length have been reported [50]. 

In general, the MUC2 expression level has been established to be ~5900-fold lower compared to MUC5AC [38]. Given mRNA expression levels are below the detection limit for visualization through in situ hybridization, it is unclear which exact conjunctival cells are responsible for MUC2 production [12]. Moreover, depending on the antibody used, different molecular weight patterns have been described [38,41]. Where both studies detect some main bands at around 250 kDa, McKenzie and colleagues further describe the presence of a high-molecular weight band of high intensity as well as a clear pattern of lower molecular weight bands, ranging from 20–84 kDa, in tears [38]. Even though it is still unclear what the role of MUC2 is at the ocular surface, it was reported that MUC2 is upregulated to compensate for the decrease in MUC5AC during an ocular surface defense response [51]. In our current study, we found a non-linear relation between MUC2 mRNA expression and protein levels. Even though the presence of MUC2 mRNA decreased during the culture period, the amount of intracellular and secreted MUC2 proteins remained quite constant. The glycosylation pattern of the hypothesized cleaved products are, however, believed to change as two additional lower bands became more prominent. It is also remarkable to note that there is not much difference between the amount of intracellular MUC2 in vivo compared to the amount stored in our ex vivo explant cultures. Hence, our results could be in line with the previous mentioned MUC2 compensation hypothesis described by Dogru [51]. 

The third gel-forming mucin, MUC5B, is known to be expressed in rat and mouse conjunctival goblet cells [52,53]. However, only a minor population of lacrimal gland cells is believed to produce MUC5B on the human ocular surface [54]. Given the low expression number and protein secretion, no studies have detected MUC5B in human tears so far [41]. There are no other published reports that detect the presence of MUC5B in isolated conjunctival cells. However, we were unable to detect MUC5B mRNA expression in conjunctival explant cultures. 

The presence of MUC6 at the ocular surface has, to our knowledge, not yet been previously investigated. MUC6 is the only gel-forming mucin lacking the cysteine-rich domains, which are important for various mucin-mucin interactions [14]. The known distribution of MUC6 encompasses the glands of the stomach, duodenal Brünner’s glands, pancreatic ducts, ilium, gall bladder, endocervix, and endometrium [14,55]. Apart from the identification of MUC6 mRNA and corresponding protein levels in isolated conjunctival cells, we were also able to detect MUC6 during the two-week explant culture period. Analogous to MUC2, a non-linear mRNA-protein relationship could be established, and the intracellular protein content of isolated conjunctival cells are similar to the cultured conjunctival cells. However, the exact function of MUC6 at the ocular surface has yet to be defined. 

To summarize, we demonstrate that both intracellular and secreted mucin levels decrease with longer times in culture; an inverse correlation that we assessed up to two weeks of explant culturing. The deteriorating goblet cell functioning could at least in part be ascribed the absence of stimuli, which could lead to dedifferentiation or transdifferentiation of the goblet cells in the explant cultures. This observation emphasizes the importance of additional functional assays besides imaging techniques to characterize and evaluate cultured conjunctival goblet cells. We further found that cultivation of explants on laminin-treated CLP-PEG hydrogel supports the production of gel-forming mucins ex vivo. Furthermore, this study identified the presence of additional gel-forming mucins (i.e., MUC5B and MUC6), previously unknown to function in the ocular surface and the yet undefined secretion of MUC2 and MUC6 by cultured conjunctival-derived cells.

## 4. Materials and Methods

### 4.1. Human Tissue

Human conjunctival tissue from 19 cadaveric donors was used in the characterization experiments, and their ages and allocations are described in Table 1. The donor’ ages ranged from 13 to 90, with an average of 65 years. Fifteen donors, who had been rejected for clinical transplantation, were obtained from the Antwerp University Tissue Bank (Antwerp, Belgium, Table 1), and processed within 48-h post-mortem. Four data-anonymized human conjunctivas were obtained from the Eversight Eye Bank (Ann Arbor Michigan, MI, USA). The isolated cadaveric tissue was placed in Optisol storage media within 18 h after death. Explant plating and culture followed within 48 h of receipt. All research was performed in accordance with the relevant guidelines and regulations. The study followed the tenets of the Declaration of Helsinki, and the use of human cadaveric donor tissue was approved by the Ethical Committee of the Antwerp University Hospital (approved EC: 11/2/12) and the Schepens Eye Research Institute and Massachusetts Eye and Ear Human Studies Internal Review Boards. The latter board decided that our use of human conjunctiva did not meet the criteria for use of human tissue. 

### 4.2. Processing of Human Conjunctival Tissue

#### 4.2.1. Isolation of Epithelial Cells from Human Conjunctiva

To obtain mRNA and intracellular proteins representing the profile of in vivo conjunctival cells, single cells were detached from their underlying connective tissue through an enzymatic dispase digestion protocol, as previously described [26]. The cellular pellet obtained was lysed using the RNeasy microkit (Qiagen), according to the manufacturer’s instructions, or using a protein lysis buffer, as specified in Section 4.6. Both lysed sample types were stored at −80 °C until further use. 

#### 4.2.2. Primary Human Conjunctival Explant Cultures (C1-–C15) 

A step-by-step protocol on the plating of bulbar conjunctiva was performed as previously described [26]. Briefly, 2 mm × 2 mm explants were cultured at the liquid air surface at 37 °C and 5% CO_2_ to initiate outgrowth. Outgrowths were submerged in culture medium consisting of supplemented keratinocyte serum-free medium, of which all the components were derived from Life Technologies (Carlsbad, CA, USA); 50 µg/mL bovine pituitary extract, 5 ng/mL recombinant human epidermal growth factor, 10 µg/mL gentamicin, and 1 µg/mL amphotericin B. Gel-forming mucin production, from mRNA expression to intracellular storage and secretion (vide infra), was evaluated after one and two weeks of culture (Table 1).

#### 4.2.3. Primary Human Conjunctival Explant Cultures (C16–19)

To define the impact of extracellular matrix hydrogels on gel-forming mucin secretion, we used a previously published method to obtain a co-cultivation of stratified squamous cells, goblet cells, and undifferentiated/stem cells [56]; starting with mincing the isolated conjunctival tissue to maintaining the cultures as described in 4.2.2. The medium was composed of Dulbecco’s Modified Eagle Medium (DMEM)/F12 (Sigma-Aldrich, St. Louis, MO, USA) supplemented with 100 µg/mL penicillin/streptomycin (Lonza, Basel, Switzerland), 1 µg/mL insulin (Sigma-Aldrich, St. Louis, MO, USA), 0.5 µg/mL hydrocortisone (Sigma-Aldrich, St. Louis, MO, USA), 2 ng/mL rat EGF (PeproTech, Cranbury, NJ, USA), and 10% human serum (Thermo Fisher Scientific, Waltham, MA, USA). The explant conjunctival cultures were photographed using an EVOS microscope (Thermo Fisher Scientific, Waltham, MA, USA).

### 4.3. Explant Culture on Extracellular Matrix Scaffolds: Collagen-like Peptide Hydrogels 

We performed explant cultures on CLP hydrogels in order to improve ex vivo mucin production and secretion. The CLP hydrogels were obtained from Ferentis (Vilnius, Lithuania). The 3D hydrogel technology including the synthesis of CLPs, conjugation with PEG maleimide, and fabrication process is described in Islam et al. [37]. We used 8.5 ± 0.2% (AVG ± STDEV, w/w) CLP-PEG scaffolds that were cross-linked using 4-(4,6-dimethoxy-1,3,5-triazin-2-yl)-4-methylmorpholinium chloride (DMTMM). Hydrogel dimensions were 15 mm and 200 ± 20 µm for the diameter and thickness, respectively. Prior to cell cultivation, the CLP-PEG hydrogels were washed twice for 30 min with phosphate buffered saline and, subsequently, with supplemented DMEM/F12 culture medium followed by an overnight incubation in freshly culture medium. Selected scaffolds were afterwards incubated with 20 µg/mL laminin (Sigma-Aldrich, St. Louis, MO, USA) at 37 °C and 5% CO_2_ for 30 min. 

### 4.4. Enzyme-linked Lectin Assay

Ulex europaeus agglutinin-1-horseradish peroxidase conjugates (UEA-1-HRP, Sigma-Aldrich, St. Louis, MO, USA) were used to measure stored and secreted mucin levels. UEA-1 binds specific carbohydrate groups present on high molecular weight mucins [22,57]. We followed the previously described ELLA protocol [58] to investigate the influence of different substrates (i.e., plastic culture ware and laminin-treated CLP-PEG hydrogels) on the absolute amount of mucin secretion. Briefly, the ELLA assay combines UEA-1-HRP conjugates (Sigma-Aldrich, St. Louis, MO, USA), Amplex red (Life Technologies, Carlsbad, CA, USA), and hydrogen peroxide (Sigma-Aldrich, St. Louis, MO, USA) to quantify the mucin concentration. Some minor modifications were implemented to address the mucin secretion as well as storage over time. The medium was changed to the supplemented keratinocyte-serum free medium, and the starvation period omitted. Both the supernatant, which contains the basal secretion, and the corresponding culture was stored at −20 °C before further processing. Mucins were solubilized in the cellular pellet by a freeze-thaw cycle and measured in the cellular lysates. Fluorescence was quantified on the Wallac 1420 VICTOR3 microplate reader (Perkin Elmer (Billerica, MA, USA); excitation 530 nm, emission 590 nm). The obtained mucin concentrations were normalized to the total protein amount, measured with Bradford (Bio-Rad, Hercules, CA, USA). 

### 4.5. RNA Extraction and Quantitative Reverse Transcription PCR

Assessment of gel-forming mucin expression (MUC2, MUC5AC, MUC5B, and MUC6) was performed as previously described in [26]. The mucin primers and corresponding template controls were purchased from Bio-Rad; qHsaCID0011696 (MUC2), qHsaCID0017663 (MUC5AC), qHsaCID0011690 (MUC5B), and qHsaCID0020103 (MUC6). Four reference genes (i.e., *CyC1* gene, *ATPB5* gene, *RPL13A* gene, and *TOP1* gene) were used for normalization purposes to compare isolated conjunctival cells and their cultured counterparts [26]. Primers for CyC1, RPL13A, and TOP1 were purchased from PrimerDesign (Eastleigh, UK) as previously described [26]. The primer used ATP5B (F—TGA-CCC-TGC-CCC-TGC-TAC-TA, R—GGA-TCT-TTT-GCA-CCC-CAC-GG) was purchased from Eurogentec (Seraing, Belgium). RT-qPCR assays were performed on a CFX96 Touch™ Real-Time PCR Detection System (Bio-Rad, Hercules, CA, USA) with following settings; an activation step of 30 s at 95 °C, 40 amplification cycles of denaturation (95 °C for 5 s) and annealing/extension (60 °C for 30 s). RT-qPCR data were analyzed with qbase+ (Biogazelle, Ghent, Belgium).

### 4.6. Western Blotting Analysis

The human lung adenocarcinoma A549 cells (ATCC CCL-185) and the human neuroblastoma SH-SY5Y cells (ATCC CRL-2266) were used as positive controls for MUC5AC/MUC6 and MUC2, respectively. The A549 cells were cultured in DMEM supplemented with 10 µg/mL gentamicin, 1 µg/mL amphotericin B, and 10% fetal bovine serum (FBS). The SH-SY5Y cells were grown in complete growth medium, containing a 1:1 mixture of DMEM and F12 medium supplemented with 10 µg/mL gentamicin, 1 µg/mL amphotericin, and 10% FBS. Prior to their mixture, DMEM and F12 medium were supplemented as well; including 1 mM sodium pyruvate, 0.1 mM nonessential amino acids, and 0.075% sodium bicarbonate for DMEM and 0.075% sodium bicarbonate F12 medium. All medium components were purchased from Life Technologies (Carlsbad, CA, USA).

The cell pellets obtained from the positive controls, isolated conjunctival-derived cells, and ex vivo cultured conjunctival cells were lysed in lysis buffer (10 mM TrisHCl, 400 mM NaCl, 1 mM EDTA, 0.1% NP40 and one tablet of Protease Inhibitor Cocktail (Roche, Basel, Switzerland)) and stored at −80 °C. As the goblet cells secrete all of their secretory granules when stimulated [59], these large, cytoplasmic, pinched-off cellular fragments were pelleted at 3000× *g* for 15 min and frozen as well. After thawing on ice, the pellets were sonicated (2 × 5 min) and centrifuged (13,000 rpm, 10 min, 4 °C) to obtain clear lysates, containing the isolated proteins. Protein concentrations were determined using the Pierce BCA protein kit (Thermo Fisher Scientific, Waltham, MA, USA). Twelve and 2.2 micrograms of respectively intracellular and secreted protein extract were reduced in 4% 2-mercaptoethanol in NuPAGE™ LDS Sample Buffer (75 °C, 10 min, Thermo Fisher Scientific, Waltham, MA, USA), run on a NuPAGE™ 3–8% Tris-Acetate Protein Gel (Thermo Fisher Scientific, Waltham, MA, USA) and transferred to an Immobilon®-P PVDF membrane (Merck Millipore, Billerica, MA, USA) during a 2-h run at 30 V and 400 mA. The Seeblue Plus2 pre-stained standard (Invitrogen (Waltham, MA, USA); LC5925) was loaded as a reference. Ponceau red validations were imaged on an Amersham Imager 680 and membranes blocked with 3% bovine serum albumin in Tris-buffered saline with 0.1% tween-20 for 1 h at room temperature. Primary (overnight, 4 °C) and secondary/β-actin (1 h, room temperature) antibody incubations were established using the SNAP id 2.0 protein detection system: MUC2 (Abcam (Cambridge, UK); ab134119, 1:1000), MUC5AC (Abcam (Cambridge, UK); ab198294, 1:20,000 dilution), MUC6 (Abcam (Cambridge, UK); ab223846, 1:1000), and β-actin (Sigma-Aldrich (St. Louis, MO, USA); 1:2500). Secondary antibodies were goat anti-rabbit (Li-Cor (Lincoln, NE, USA); 1:10,000) or goat anti-mouse (Li-Cor (Lincoln, NE, USA); 1:10,000). Protein bands were imaged using the Odyssey imaging system (Li-Cor, Lincoln, NE, USA). When two types of mucins were detected on the same membrane, the antibodies were stripped using the Re-blot strong solution (Merck Millipore, Billerica, MA, USA) according to the manufacturer’s instructions. The AIDA Image Analysis software (Elysia s.a., Angleur, Belgium) was used for intensity quantifications. The intensity was normalized to the total protein content and depicted as the intensity per loaded µg of protein.

### 4.7. Histology and Immunocytochemistry

The ex vivo conjunctival cultures were fixed in 4% paraformaldehyde at 4 °C for 20 min, followed by a triple washing step with phosphate buffered saline. Stored mucin was detected using the PAS staining kit (Merck Millipore, Billerica, MA, USA), according to the manufacturer’s instructions. Presence of MUC5AC (goblet cell marker) and MUC1 as well as CK13 (i.e., epithelial cell marker) were investigated by immunocytochemistry. Briefly, fixed cultures were permeabilized with 1% triton X-100 blocking buffer (30 min) and primary antibodies against MUC1 (Abcam (Cambridge, UK); ab15481, 1:200 dilution), MUC5AC (Abcam (Cambridge, UK); ab198294, 1:500 dilution), and CK13 (Abcam (Cambridge, UK); ab92551, 1:500 dilution) were incubated overnight at 4 °C. Cy3-conjungated donkey-anti-rabbit antibody (Jackson ImmunoResearch, Cambridge, UK) was added for 2 h at 4 °C, followed by a nuclear counterstain using 4′,6-diamidino-2-phenylindole (DAPI) for 1 min at room temperature. Samples were mounted in citifluor and imaged on an Ultra*VIEW* VoX dual spinning disk confocal system (PerkinElmer, Billerica, MA, USA).

### 4.8. Statistical Analysis

GraphPad Prism 5 software (GraphPad software, San Diego, CA, USA) was used for statistical and graphical purposes. Different tests were considered as found appropriate with normality testing and are specified in the result section. The Shapiro–Wilk test was chosen as it has been identified as the most powerful normality test for small and moderate sample sizes [60]. Paired measures were analyzed with a Wilcoxon test or a paired *t*-test (e.g., comparison between stored and secreted mucin content of one conjunctival outgrowth culture). Unpaired measures were evaluated with either an unpaired *t*-test, Mann–Whitney U test, One-Way ANOVA or Kruskal–Wallis test (e.g., different cultures of the same donors over time), as indicated. The CNRQ values representing the gel-forming mucin mRNA levels were obtained using the qbase+ software (Biogazelle, Ghent, Belgium). Statistical analysis was performed using JMP PRO 15 software (SAS Institute, North Carolina, NC, USA) and the values underwent logarithmic transformation. Statistically significant changes in mRNA expression were identified by data fitting in a mixed effect model, implementing DIC as a continuous variable and the donor number as random effect. The accompanying fixed effect test identified a potential slope in the mRNA expression over time. 

## Figures and Tables

**Table 1:** Donor characteristics and experimental involvement.

Donor	Age	Country	Experiments	Specifications
C1	58	Belgium	RT-qPCR	0 and 14 DIC
C2	78	Belgium	RT-qPCR	0 and 14 DIC
C3	70	Belgium	RT-qPCR	0 and 14 DIC
C4	88	Belgium	RT-qPCR	0 and 14 DIC
C5	69	Belgium	RT-qPCR	6, 8, 12, and 14 DIC
C6	84	Belgium	RT-qPCR	0, 4, 6, 8, 11, and 13 DIC
C7	90	Belgium	RT-qPCR	0, 4, 6, 8, 11, and 13 DIC
C8	79	Belgium	RT-qPCR	6, 8, 10, 12, and 14 DIC
C9	81	Belgium	RT-qPCR	8, 11, and 14 DIC
C10	63	Belgium	RT-qPCR	6, 8, 10, 12, and 14 DIC
C11	52	Belgium	ICC	Illustration CK13-staining (8 and 14 DIC)
C12	77	Belgium	ELLA	Evolution over time (7 and 14 DIC)
			Western blot	Stored (0, 7, and 14 DIC) and secreted mucin (14 DIC)
			Histochemistry	Illustration PAS-staining (14 DIC)
			ICC	Illustration MUC1-staining (14 DIC)
C13	42	Belgium	ELLA	Evolution over time (7 and 14 DIC)
			Western blot	Stored (0, 7, and 14 DIC) and secreted mucin (14 DIC)
			ICC	Illustrative image of MUC1-staining (7 DIC)
C14	68	Belgium	ELLA	Evolution over time (7 and 14 DIC)
C15	64	Belgium	Western blot	Stored (0, 7, and 14 DIC) and secreted (7 and 14 DIC)
			ICC	Illustration MUC5AC-staining (7 and 14 DIC)
C16	38	USA	Micrographs	Illustration of outgrowth on plastic and ECM hydrogels
C17	13	USA	ELLA	Culture’s plastic vs ECM hydrogels
C18	68	USA	ELLA	Culture’s plastic vs ECM hydrogels
C19	57	USA	ELLA	Culture’s plastic vs ECM hydrogels

CK, cytokeratin; DIC, days in culture; ECM, extracellular matrix; ELLA, enzyme-linked lectin assay; ICC, immunocytochemistry; MUC, mucin; PAS, Periodic Acid–Schiff; RT-qPCR, Quantitative reverse transcription PCR; USA, United States of America

## Data Availability

The data presented in this study are available on request from the corresponding author. The data are not publicly available.
